# Large variation in participant eligibility criteria used in plantar heel pain research studies - a systematic review

**DOI:** 10.1186/s13047-022-00573-0

**Published:** 2022-09-09

**Authors:** Henrik Riel, Melanie Louise Plinsinga, Eamonn Delahunt, Martin Bach Jensen, Karl B. Landorf, Marienke van Middelkoop, Edward Roddy, Michael Skovdal Rathleff, Bill Vicenzino, Jens Lykkegaard Olesen

**Affiliations:** 1 Center for General Practice at Aalborg University, Fyrkildevej 7, 9220 Aalborg East, Denmark; 2 The University of Queensland, School of Health and Rehabilitation Sciences: Physiotherapy: Sports Injury Rehabilitation and Prevention for Health, St. Lucia, QLD 4072 Australia; 3 Menzies Health Institute Queensland, Griffith University, Brisbane, 4111 QLD Australia; 4 Institute for Sport and Health, University College Dublin, Dublin, Ireland; 5 School of Public Health, Physiotherapy and Sports Science, University College Dublin, Dublin, Ireland; 6 Discipline of Podiatry, School of Allied Health, Human Services and Sport, La Trobe University, Bundoora, 3086 Australia; 7 La Trobe Sport and Exercise Medicine Research Centre, La Trobe University, Bundoora, 3086 Australia; 8 Department of General Practice, Erasmus MC Medical University Center Rotterdam, Rotterdam, The Netherlands; 9 School of Medicine, Keele University, Staffordshire, ST5 5BG UK; 10 Haywood Academic Rheumatology Centre, Midlands Partnership NHS Foundation Trust, Stoke-on-Trent, ST6 7AG UK; 11 Department of Health Science and Technology, Faculty of Medicine, Aalborg University, Aalborg, Denmark; 12 Department of occupational therapy and physiotherapy, Aalborg University Hospital, Hobrovej 18-22, 9100 Aalborg, Denmark

**Keywords:** Plantar fasciitis, Heel spur syndrome, Participant characteristics, Heterogeneity, Plantar heel pain

## Abstract

**Introduction:**

Variable eligibility criteria across studies on plantar heel pain may result in compromising the generalisability of meta-analyses when heterogeneity is not accounted for. We aimed to explore: (i) heterogeneity of participant eligibility criteria in studies that have investigated plantar heel pain, and (ii) associations between key eligibility criteria and the characteristics of the participants included in the study.

**Methods:**

In this systematic review with narrative synthesis, we extracted participant eligibility criteria, and participants’ age, body mass index (BMI), symptom duration and pain level from published studies on plantar heel pain. We performed a content analysis of criteria and aligned overarching criteria to the International Classification of Functioning, Disability and Health (ICF). We pooled studies that used the same thresholds for participant eligibility criteria into sub-groups. We also pooled and reported studies that did not have any eligibility criteria for the quantitative characteristics to use their data for reference values and pooled studies that did not have any eligibility criteria for the characteristics as reference.

**Results:**

Two hundred and fourteen articles were included. The most reported participant eligibility criteria (as aligned to the ICF) related to body structures/function and personal factors. Age, BMI, symptom duration and pain level were used with various ranges and/or thresholds across studies (age was reported in 23 different ways across 97 studies; BMI 7/13; symptom duration 14/100; and pain level 8/31). When eligibility criteria included thresholds close to the reference value of a participant characteristic, characteristics were associated with criteria (e.g., younger participants when an upper age threshold was used).

**Conclusion:**

Participant eligibility criteria in studies on plantar heel pain vary widely; studies differed substantially in their use of quantitative thresholds. Participant characteristics of samples in studies were associated with the criteria used. This study emphasises a need for adjusting for participant heterogeneity in systematic reviews to improve their validity.

**Supplementary Information:**

The online version contains supplementary material available at 10.1186/s13047-022-00573-0.

## Background

Plantar heel pain (PHP) has a yearly prevalence of 2.4 to 6.5 per 1000 registered patients in general practice and affects approximately 8 to 31% of runners [1–4]. The underlying aetiology of PHP is unknown, but is believed to be a consequence of tissue overload, via a high body mass index (BMI) and/or prolonged weight-bearing exercise [5, 6].

We previously highlighted a lack of consistency across published literature in the terminology used to describe pain in the plantar surface of the heel (e.g. plantar fasciitis, plantar fasciopathy, heel spur syndrome, and many more) [7]. This issue with inconsistent terminology may be further compounded by heterogeneity in the participant eligibility criteria used in research studies on PHP, as has been reported in the back pain literature [8]. Variability in eligibility criteria across studies on PHP may result in heterogeneous samples, which may compromise the generalisability of meta-analyses investigating the efficacy or effectiveness of interventions when heterogeneity is not accounted for. Using recommended participant eligibility criteria that are based on robust research findings and consensus may minimise this issue [9–12]. For example, participant characteristics such as age, sex, whether symptoms are unilateral or bilateral, and duration of symptoms have all been found to be associated with prognosis [13, 14]. However, before recommendations for participant eligibility criteria for research studies related to PHP are developed, it is important to determine the extent of the heterogeneity amongst published studies and explore if selected criteria are associated with participant characteristics.

The aim of this systematic review was to explore two issues related to studies that have investigated PHP: (i) heterogeneity of participant eligibility criteria, and (ii) associations between key eligibility criteria and the characteristics of the participants included in the study.

## Methods

This systematic review with narrative synthesis is reported according to the Preferred Reporting Items for Systematic Reviews and Meta-analyses (PRISMA) guidelines and was prospectively registered in the International Prospective Register of Systematic Reviews (PROSPERO); registration no. CRD42018107439 available from https://www.crd.york.ac.uk/prospero/display_record.php?ID=CRD42018107439 [15].

### Search strategy and eligibility criteria

We included published prospective or cross-sectional experimental and observational studies with adult participants (age > 18 years) with PHP, heel spur syndrome, plantar fasciitis, plantar fasciopathy, plantar fasciosis, painful heel syndrome or calcaneodynia. We excluded studies that were undertaken in populations with differential diagnoses of PHP such as spondyloarthritis, fat-pad atrophy, proximal plantar fibroma, calcaneal stress fracture, insertional Achilles tendinopathy, and non-painful heel spurs. In addition, we excluded retrospective studies (studies in which outcomes of interest were collected before the start of the study). We conducted literature searches in the following databases: PubMed, Embase, Cochrane Central Register of Controlled Trials (CENTRAL), Web of Science (WoS), CINAHL, and Physiotherapy Evidence Database (PEDro). The search was limited to studies published between January 1st, 1998 and March 4th, 2019, and there were no language restrictions. The search strategy was developed in collaboration with a librarian with expertise in designing such strategies for systematic reviews and was adapted to fit each database. We used the following search terms: plantar fasciitis, heel spur*, heel pain, policeman’s heel, calcaneal spur*, calcaneal pain, plantar fasci*, and plantar aponeuros*.

### Study selection

Two authors (HR and MLP) independently screened the studies that were retrieved from the search using Covidence (Covidence systematic review software, Veritas Health Innovation, Melbourne, Australia). If a study was published in a language that the two authors were unable to understand, they would consult another researcher with sufficient language proficiency in that language. After screening titles and abstracts, studies were grouped into: (i) randomised trials, and (ii) non-randomised study designs. As we expected a large number (> 500) of studies to be eligible, we decided a priori to randomly select 50% of the randomised trials stratified by year for full-text screening, with the objective of including a representative sample of trials. Trials that were described as randomised, but which were subsequently found on closer scrutiny to be non-randomised were moved to the pool of non-randomised study designs. After the inclusion of randomised trials, we screened (via full-text) the same number of studies with non-randomised designs to arrive at a final sample of studies that included an equal proportion of randomised trials (50%) and non-randomised study designs (50%). We used a weighted random selection based on the year the included randomised trials were published to match the yearly distribution of the non-randomised study designs. When a study with a non-randomised design was excluded during screening, we included a new study published in the same year as the excluded study and continued this process until the number of included studies with non-randomised designs matched that of the randomised trials. If any disagreements about inclusion arose during screening and consensus could not be reached between HR and MLP, a third author (JLO) was consulted to make the final decision.

### Data extraction

Data extraction was performed independently by HR and MLP. Any disagreements were resolved by discussion. Data extraction forms were developed a priori and included: terminology, setting, method of recruitment, country, interventions, eligibility criteria, number of participants, age, height, weight, BMI, symptom duration, pain intensity, and physical activity level. If studies only reported height and mass, we calculated BMI as kg/m^2^. In studies with multiple groups, we combined the groups and calculated a weighted mean and SD for any participant eligibility criterion that was measured on a continuous scale. If studies reported the median and range, we estimated the mean and SD [16]. In case of missing data or discrepancy between criteria and participant characteristics reported, we contacted the authors via e-mail for clarification. The validity of the diagnosis was assessed independently by HR and MLP and evaluated as either high (based on physical examination and/or imaging), low (based on patients’ self-reported symptoms), or unclear (unspecified). This classification was based on clinical guidelines that recommend physical examination [17].

### Data synthesis

To analyse the participant eligibility criteria and evaluate the extent of variation (Aim 1), we considered criteria qualitative data and undertook a content analysis in NVivo version 11 (QSR International, Melbourne, Australia). One author (HR) initially divided the criteria into overarching criteria and then the final content analysis was performed together with another author (JLO). As an example, measures of pain were first extracted as worst pain, first-step pain, or average pain over time, forming the overarching criterion ‘pain’. We calculated the number of studies that used criteria within a given overarching criterion, however one study may have had several criteria within the same overarching criterion. For example, several differential diagnoses and comorbidities may have been used as exclusion criteria in a single study, but this study was only counted as one within the overarching criterion called ‘other diagnoses’. To use an overarching framework, we aligned overarching criteria to the International Classification of Functioning, Disability and Health (ICF). Thereby, participant eligibility criteria were classified as being related to either participation, body structures/function, environmental factors, activities, or personal factors [18]. After the content analysis, all authors decided by consensus if there was a high variability in the participant eligibility criteria used. In this process, we considered the number of overarching criteria within the ICF framework as well as the between-study variation of how the same criterion was used. For example, the criterion of ‘pain’ has sub-criteria such as worst pain, first-step pain, and average pain, and these sub-criteria may have different eligibility thresholds on a 0 to 10 cm Visual Analogue Scale (VAS).

To test the association between the criteria of age, BMI, symptom duration and pain intensity with the corresponding participant characteristics (Aim 2), we separated studies into those that specified these as selection criteria from those that did not. Those that pre-specified criteria were further pooled into those that used the same ranges and thresholds for these measures. We also pooled studies that did not include these participant characteristics in their eligibility criteria but used these as a reference value for each of the characteristics. For example, if a study did not use age as a criterion, but still reported participants’ age, this study was pooled into a reference sub-group for age. Based on the sample size of the studies, we calculated a weighted mean of the reported characteristics within each of the sub-groups and compared these between groups. We did not use any inferential statistics as weighted means were used so the common assumptions were not met in our design. Instead, we present the raw data in plots to enable the reader to interpret the data.

## Results

**Fig. 1:**
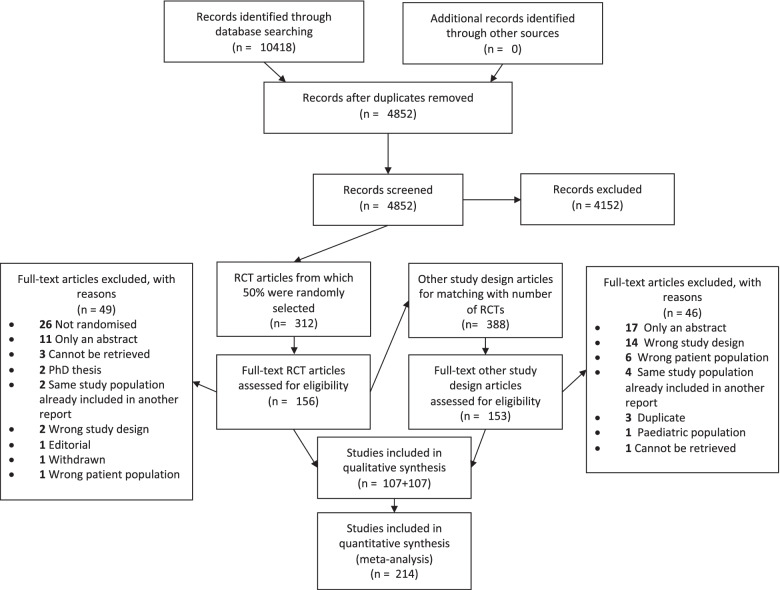
PRISMA flowchart

We identified 10,418 citations through our search, which were reduced to 4852 after removing duplicates (Fig. 1). During the screening of titles and abstracts, we identified 700 studies that were eligible for full-text screening. Of these, 312 were initially considered randomised trials and we randomly selected 156 of these for full-text screening. We subsequently included 107 trials that met the criteria. Following this, 107 randomly selected non-randomised studies that met the criteria were included. A list of included studies may be found in Supplementary file 1.

The 214 studies included 12,330 participants with PHP. The most used terminology to describe the condition was plantar fasciitis (*n* = 153), followed by plantar heel pain (*n* = 26) and plantar fasciopathy (*n* = 9). Most studies originated from the USA (*n* = 34), Germany (*n* = 24), and Turkey (*n* = 24). The validity of the diagnosis was evaluated as being high in 149/214 (69.6%) studies.

### Aim 1

As aligned to the ICF framework, the most used participant eligibility criteria related to body structures/function and personal factors (Table 1). Only one study used a criterion that related to environmental factors. The most common overarching criterion was exclusion due to diagnoses other than PHP (used by 170/214 (79%) studies), followed by criteria concerning PHP traits such as symptom duration, presence of pain on palpation, pain intensity, pain location, and pain description (used by 165/214 (77%) studies). Age was a criterion in 97/214 (45%) studies and was quantified in 23 different ways. BMI was a criterion in 13/214 (6%) studies and was quantified in 7 different ways. Symptom duration was a criterion in 100/214 (47%) studies and was quantified in 14 different ways. Pain was a criterion in 50/214 (23%) studies and was defined as: ‘after taking a few steps’, ‘during activity’, ‘first-step pain’, ‘worst pain’, and simply ‘pain’ without any further clarification. Each of these pain sub-criteria used different eligibility thresholds for pain. For example, 14 studies used ‘first-step pain’ as a criterion but quantified it in 8 different ways. ‘Pain’ was the most common criterion for pain intensity and was used by 31/50 (62%) studies and quantified in 8 different ways. Due to the heterogeneity in the use of pain as a criterion and in order to be able to make meaningful comparisons between sub-groups, we sub-grouped ‘pain’ with no further clarification when we explored associations between the criterion used and participant characteristics.

**Table 1:** Grouping of participant eligibility criteria according to ICF framework

**Participation**	**Body structures/function**
Occupation related (13–4) Other research participation (2–4)	Other diagnoses (90–80) Condition traits (92–73) ▪ Symptom duration (66–44) ▪ Pain intensity (34–16) Anatomy (37–37) ▪ BMI (7–6) Contraindication (36–17) Functioning (20–13) Cognition (14–10) Diagnostic imaging (10–13) Mental health (5–2) Treatment eligibility (3–2) General health (1–2) Diagnostic nerve block positivity (0–1)
**Environmental factors**	**Activities**
Participation decided by investigator (1–0)	Physical activity (4–3)
**Personal factors**	
Age (64–33) History of surgery and treatments NOT specifically for PHP (57–34) Previous treatment for PHP (56–35) Pregnancy (50–20) Medicine intake and injections not necessarily for PHP (48–15) Having undergone unsuccessful preceding treatment as inclusion (38–29) Compliance with participation (18–6) Willingness (14–9) Language proficiency (6–7) Substance abuse (3–3) Planned treatment (2–0)	

Note: numbers in the table represent the number of randomised studies with one or more criteria within an overarching criterion – the number of non-randomised study designs with one or more criteria within an overarching criterion

### Aim 2

**Fig. 2:**
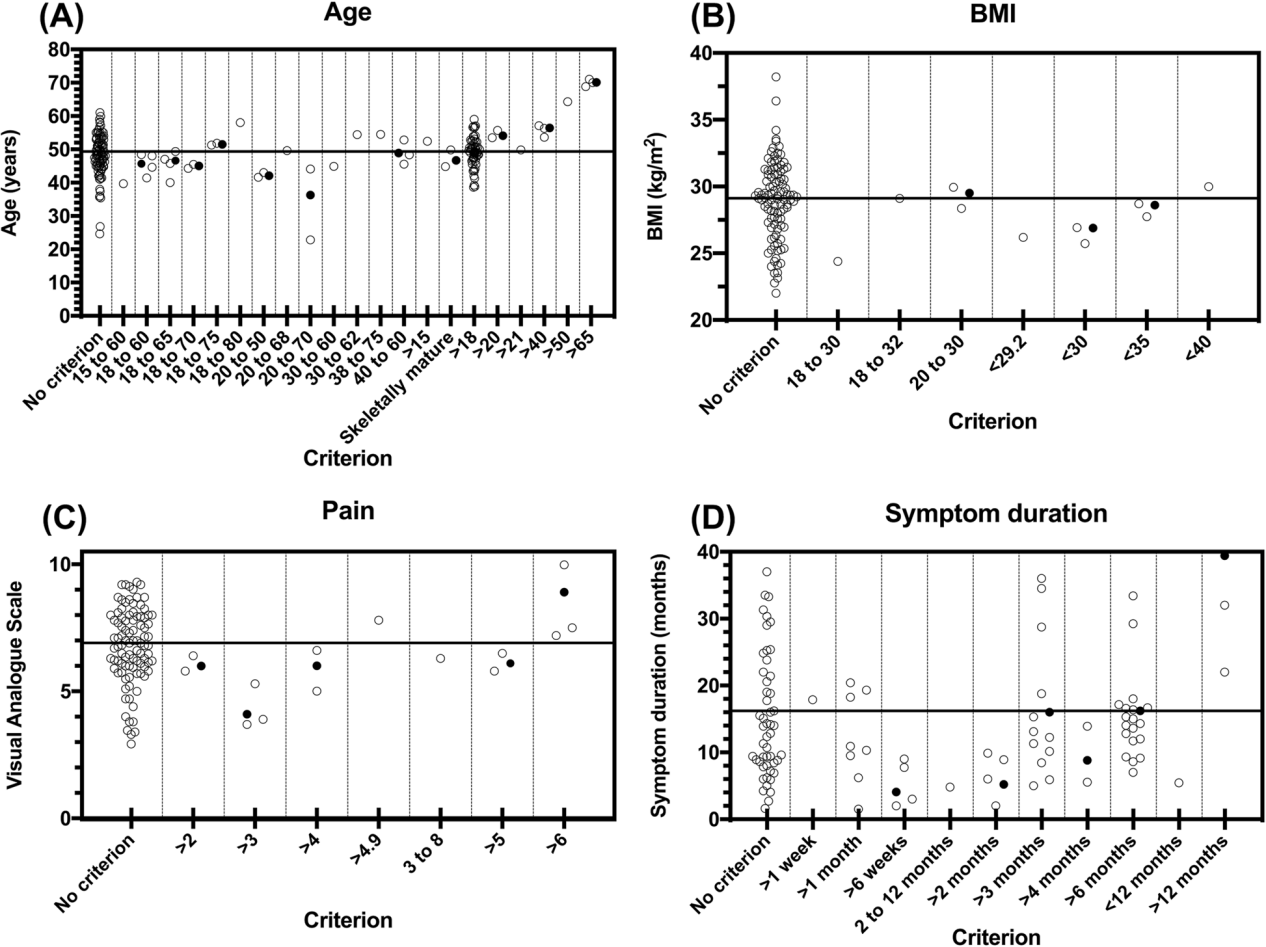
**A** Age (years) data from each individual study according to the age criterion sub-group. Circles depict age as reported by the study and the solid dots depict the weighted mean within the sub-group. The solid horizontal line illustrates the reference value (49.2 years), which is the weighted mean among studies that reported the value but did not have an age criterion. **B** BMI (kg/m^2^) data from each individual study according to the BMI criterion sub-group. Circles depict BMI as reported by the study and the solid dots depict the weighted mean within the sub-group. The solid horizontal line illustrates the reference value (29.2 kg/m^2^), which is the weighted mean among studies that reported the value but did not have a BMI criterion. **C** Pain (VAS) data from each individual study according to the pain criterion sub-group. Circles depict pain as reported by the study and the solid dots depict the weighted mean within the sub-group. The solid horizontal line illustrates the reference value (6.9 VAS), which is the weighted mean among studies that reported the value but did not have a pain criterion. **D** Symptom duration (months) data from each individual study according to the symptom duration criterion sub-group. Circles depict symptom duration as reported by the study and the solid dots depict the weighted mean within the sub-group. The solid horizontal line illustrates the reference value (16.2 months), which is the weighted mean among studies that reported the value but did not have a symptom duration criterion

Data relating to Aim 2 are presented in Supplementary file 2. Age was reported, but not used as a criterion in 100/214 (47%) studies. The weighted mean age of participants was 49.2 years. BMI was reported, but not used as a criterion in 103/214 (48%) studies. The weighted mean BMI was 29.2 kg/m^2^. Symptom duration was reported, but not used as a criterion in 53/214 (25%) studies, with a weighted mean duration of symptoms of 16.2 months. Pain intensity was reported, but not used as a criterion in 94/214 (44%) studies. The weighted mean pain intensity was 6.9 cm as measured on a 10 cm VAS. Overall, participant eligibility criteria with ranges that were close to being equally distributed around the reference value (obtained from studies that did not specify it as a selection criterion) did not affect the characteristics of recruited participants (Fig. 2). For example, Fig. 2A shows the criterion age, where three studies used the range 40 to 60 years as an eligibility criterion. The mean age (participant characteristic) found in these studies was 0.3 years lower than the reference value (49.2 years). Three studies that used the same lower limit of age, but in contrast to the three aforementioned studies did not use an upper limit, reported a mean age 7.2 years higher than the reference value.

## Discussion

In this review of the eligibility criteria employed by clinical studies of PHP, we observed substantial variability in the participant eligibility criteria across included studies, as well as how these criteria were used. Second, across individual studies, participant eligibility criteria were associated with participant characteristics, highlighting the impact that participant eligibility criteria can have on the characteristics of the recruited study population.

The included studies used many different participant eligibility criteria, as well as different quantitative ranges and/or thresholds for the criteria. As an example, age was used by 97/214 studies but quantified in 23 different ways. Therefore, seemingly similar overarching criteria are used very differently across studies. Based on our findings, it appears relevant to align eligibility criteria with the research question. Limiting certain characteristics (e.g. BMI) through the use of eligibility criteria should only be applied if researchers have a clear rationale for it. Including specific limits without strong rationales may hamper inclusion in meta-analysis as it can violate the assumption of homogeneity between samples [12]. Another criterion that may be less relevant when not linked to the research question is pain level. For example, four studies used a pain level > 6 cm VAS despite not explicitly investigating a patient group more affected by the condition than others. It is sensible, however, to use a minimal pain level as an eligibility criterion in longitudinal trials investigating a treatment to avoid a floor effect, but the pain level should reflect that seen in everyday clinical practice [19].

There appears to be an association between thresholds and cut-offs applied to participant eligibility criteria and the associated characteristics of participants. However, many thresholds seem to have limited impact as the average value reported is like that of studies that did not use a criterion to restrict this characteristic. For example, the average age among participants in studies that did not use age as a specific participant eligibility criterion was 49.2 years and the average age among participants in studies using age from 40 to 60 years as a specific participant eligibility criterion was 48.9 years. The variation might be less, but the average is almost the same. If researchers truly want to include a very specific participant type that differs from the average, it is important to use a cut-off that shifts that criterion away from the average. With age, for example, it appears that using a lower limit of 40 years with no upper limit is required to include participants older than the average participant. The closer either a lower or upper limit of any given characteristic is to the population average, the more likely it is to affect the profile of the participants included.

Even if participants’ characteristics are affected by the choice of participant eligibility criteria, any important differences in age, BMI, symptom duration and baseline pain level remain unknown. This lessens the relevance of manipulating the study population characteristics through the application of specific participant eligibility criteria. In prospective studies that investigate change over time, age and symptom duration may be of importance as these characteristics have been found to have prognostic value [13]. Therefore, the choice of age span may affect study outcomes in terms of treatment effect, as patients younger than 40 years of age have a worse prognosis compared to older patients [13].

Not only may eligibility criteria affect participants’ characteristics, but they may also have implications for the validity of systematic reviews with or without meta-analyses. These evidence syntheses rely on the basic assumption that studies can be pooled because there is homogeneity of the samples between studies [20]. Efforts have been made in the past to develop recommendations on how to address heterogeneity in systematic reviews [12]. This may be taken into account by pre-planned subgroup analyses or by ﻿conducting a meta-analysis with a fixed-effects or random-effects model [21]. We believe that our results support the need for such approaches as the use of participant eligibility criteria appear to be associated with participant characteristics. This could improve the validity and applicability of the findings of systematic reviews across research fields. To our knowledge, there is only one review of the heterogeneity of criteria used, which was in back pain [8]. That review also found variation in the number and type of eligibility criteria from one study to another. This leads us to speculate that this issue applies to other conditions besides PHP and that researchers should become increasingly aware of the impact the selection of participant eligibility criteria may have on the recruited participants. Unless researchers seek to include a specific sub-group of the patient population, caution should be made when using restrictive eligibility criteria.

There is no single correct approach to selecting participant eligibility criteria – but rather, they should align with the research question. Too many criteria may hamper generalisability to the wider population with the condition [22]. In oncology research, researchers have been advised to broaden participant eligibility criteria as they have become too restrictive [23]. We believe that our results do not support the use of a recommended set of participant eligibility criteria for PHP research, but they may be used to guide decisions for those who want to include a sub-group of patients (e.g. younger, active or non-overweight individuals).

A limitation of our review is the reliance on characteristics reported by authors, even if they do not match with their stated selection criteria. For example, one study reported that one of their sub-groups had an average BMI above 30 ﻿kg/m^2^ when they pre-specified a selection criterion of having a BMI of < 30 kg/m^2^. Further, the large number of different criteria sub-groups (Fig. 2), which were created because of the variability of thresholds and ranges, led to these sub-groups having only a few studies included in them. This makes outlier studies more impactful. Another limitation is that we included only 50% of studies published over the time period of our search. There are studies which we did not include that potentially could have led to slight changes to the results.

## Conclusion

This systematic review found that there is substantial variability in participant eligibility criteria used in published PHP studies, both in the specific criteria that they use and the thresholds and/or the cut-offs applied to these criteria. The careful selection of participant eligibility criteria is important due to the association between which criteria are used and the corresponding participant characteristics of recruited samples. Several previous studies used criteria that may have unintentionally led to the inclusion of a sub-group of individuals that may not be representative of the wider PHP population, which hampers the generalisability of their results and the generalisability of the findings of systematic reviews in which they are included.

## Supplementary Information

**Additional file 1.**

**Additional file 2.**

## Data Availability

Data will be made available upon reasonable request.

## Abbreviations

BMI: Body mass index
ICF: International Classification of Functioning, Disability and Health
PHP: Plantar heel pain
SD: Standard deviation
VAS: Visual analogue scale
