# Supplementary material for: Large variation in participant eligibility criteria used in plantar heel pain research studies - a systematic review
Source: J Foot Ankle Res. 2022 Sep 9;15:69. doi: 10.1186/s13047-022-00573-0 (PMC9461187; doi:10.1186/s13047-022-00573-0)
Supplement: Supplementary file 1 — Additional file 1. [file 13047_2022_573_MOESM1_ESM.pdf]

| Study | Title |
|---|---|
| Abd El Salam 2011 | Low-dye taping versus medial arch support in managing pain and pain-related disability in patients with plantar fasciitis |
| Abt 2002 | Shock wave therapy for recalcitrant plantar fasciitis with heel spur: prospective randomized placebo-controlled double-blind study |
| Acosta-Olivo 2017 | Plantar Fasciitis-A Comparison of Treatment with Intralesional Steroids versus Platelet-Rich Plasma A Randomized, Blinded Study |
| Ahmad 2017 | Treatment of Plantar Fasciitis With Botulinum Toxin: A Randomized, Controlled Study |
| Ahmadzadeh 2019 | Effect of dose of the corticosteroid injected locally on inflammatory diseases |
| Ajimsha 2014 | Effectiveness of myofascial release in the management of plantar heel pain: a randomized controlled trial |
| Akinoglu 2017 | Comparison of the Acute Effect of Radial Shock Wave Therapy and Ultrasound Therapy in the Treatment of Plantar Fasciitis: A Randomized Controlled Study |
| Al-Bluwi 2011 | Efficacy of EZStep in the management of plantar fasciitis: a prospective, randomized study |
| Alotaibi 2015 | Effect of Monophasic Pulsed Current on Heel Pain and Functional Activities caused by Plantar Fasciitis |
| Babaei-Ghazani 2019 | Comparison of Ultrasound-Guided Local Ozone (O2-O3) Injection vs Corticosteroid Injection in the Treatment of Chronic Plantar Fasciitis: A Randomized Clinical Trial |
| Baldassin 2009 | Effectiveness of Prefabricated and Customized Foot Orthoses Made From Low-Cost Foam for Noncomplicated Plantar Fasciitis: A Randomized Controlled Trial |
| Ball 2013 | Steroid injection for inferior heel pain: a randomised controlled trial |
| Basford 1998 | A randomized controlled evaluation of low-intensity laser therapy: Plantar fasciitis |
| Bonanno 2011 | Pressure-relieving properties of various shoe inserts in older people with plantar heel pain |
| Canyilmaz 2015 | Prospective Randomized Comparison of the Effectiveness of Radiation Therapy and Local Steroid Injection for the Treatment of Plantar Fasciitis |
| Catal 2017 | Endoscopic Plantar Fasciotomy; Deep Fascial Versus Superficial Fascial Approach: A Prospective Randomized Study |
| Chan 2015 | The effects of the application of low-dye taping on paretic side plantar pressure among patients with plantar fasciitis |
| Chetri 2016 | A Comparitive Study on Effectiveness of Taping with Iontophoresis and Taping Alone in Chronic Plantar Fascitis |
| Chow 2007 | Comparison of different energy densities of extracorporeal shock wave therapy (ESWT) for the management of chronic heel pain |
| Cinar 2017 | Low-level laser therapy in the management of plantar fasciitis: a randomized controlled trial |
| Cinar 2018 | Combination Therapy Versus Exercise and Orthotic Support in the Management of Pain in Plantar Fasciitis: A Randomized Controlled Trial |
| Cleland 2009 | Manual Physical Therapy and Exercise Versus Electrophysical Agents and Exercise in the Management of Plantar Heel Pain: A Multicenter Randomized Clinical Trial |
| Costantino 2014 | Cryoultrasound therapy in the treatment of chronic plantar fasciitis with heel spurs. A randomized controlled clinical study |
| Crawford 1999 | Steroid injection for heel pain: evidence of short-term effectiveness. A randomized controlled trial |
| Das 2015 | The Effects of Interferential Therapy for Industrial Workers in Case of Plantar Fasciitis |
| DiGiovanni 2006 | Plantar fascia-specific stretching exercise improves outcomes in patients with chronic plantar fasciitis. A prospective clinical trial with two-year follow-up |
| Dogramaci 2010 | Intracorporeal pneumatic shock application for the treatment of chronic plantar fasciitis: a randomized, double blind prospective clinical trial |
| Eftekharsadat 2016 | Dry needling in patients with chronic heel pain due to plantar fasciitis: A single-blinded randomized clinical trial |

| | |
|---|---|
| Ermutlu 2018 | Thickness of plantar fascia is not predictive of functional outcome in plantar fasciitis treatment |
| Ersen 2018 | A randomized-controlled trial of prolotherapy injections in the treatment of plantar fasciitis |
| Fernandez-Rodriguez 2018 | Prospective Randomized Trial of Electrolysis for Chronic Plantar Heel Pain |
| Fouda 2016 | Effect of Low Energy Versus Medium Energy Radial Shock Wave Therapy in the Treatment of Chronic Planter Fasciitis |
| Ge 2017 | Dry cupping for plantar fasciitis: a randomized controlled trial |
| Gerdesmeyer 2008 | Radial extracorporeal shock wave therapy is safe and effective in the treatment of chronic recalcitrant plantar fasciitis: Results of a confirmatory randomized placebo-controlled multicenter study |
| Gollwitzer 2007 | Extracorporeal shock wave therapy for chronic painful heel syndrome: a prospective, double blind, randomized trial assessing the efficacy of a new electromagnetic shock wave device |
| Grecco 2013 | One-year treatment follow-up of plantar fasciitis: radial shockwaves vs. conventional physiotherapy |
| Guner 2013 | Effectiveness of Local Tenoxicam Versus Corticosteroid Injection for Plantar Fasciitis Treatment |
| Gurcay 2017 | Shall We Inject Superficial or Deep to the Plantar Fascia? An Ultrasound Study of the Treatment of Chronic Plantar Fasciitis |
| Haake 2003 | Extracorporeal shock wave therapy for plantar fasciitis: randomised controlled multicentre trial [with consumer summary] |
| Hammer 2003 | Extracorporeal shock wave therapy (ESWT) in patients with chronic proximal plantar fasciitis: A 2-year follow-u |
| Harlapur 2010 | Comparison of myofascial release and positional release therapy in plantar fasciitis -- a clinical trial |
| Hawamdeh 2016 | The short-term effect of extracorporeal shock wave in treating plantar fasciitis: RCT |
| Hsi 1999 | In-shoe pressure measurements with a viscoelastic heel orthosis |
| Ibrahim 2010 | Chronic Plantar Fasciitis Treated with Two Sessions of Radial Extracorporeal Shock Wave Therapy |
| Karimzadeh 2017 | Autologous whole blood versus corticosteroid local injection in treatment of plantar fasciitis: A randomized, controlled multicenter clinical trial |
| Katzap 2018 | Additive Effect of Therapeutic Ultrasound in the Treatment of Plantar Fasciitis: a Randomized Controlled Trial |
| Kim 2015 | Effectiveness of polydeoxyribonucleotide injection versus normal saline injection for treatment of chronic plantar fasciitis: a prospective randomised clinical trial |
| Kudo 2006 | Randomized, placebo-controlled, double-blind clinical trial evaluating the treatment of plantar fasciitis with an extracorporeal shockwave therapy (ESWT) device: A North American confirmatory study |
| Kumar 2017 | Efficacy of Myofascial Release Technique in Chronic Plantar Fasciitis: A Randomized Controlled Trial |
| Kumnerddee 2012 | Efficacy of Electro-Acupuncture in Chronic Plantar Fasciitis: A Randomized Controlled Trial |
| Labek 2005 | Influence of local anesthesia and energy level on the clinical outcome of extracorporeal shock wave-treatment of chronic plantar fasciitis - A prospective randomized clinical trial |
| Lai 2018 | Ultrasonography and clinical outcome comparison of extracorporeal shock wave therapy and corticosteroid injections for chronic plantar fasciitis: A randomized controlled trial |
| Landorf 2006 | Effectiveness of foot orthoses to treat plantar fasciitis: a randomized trial |
| Lee 2007 | Intralesional autologous blood injection compared to corticosteroid injection for treatment of chronic plantar fasciitis. A prospective, randomized, controlled trial |
| Lohrer 2010 | Comparison of Radial Versus Focused Extracorporeal Shock Waves in Plantar Fasciitis Using Functional Measures |
| Lynch 1998 | Conservative treatment of plantar fasciitis a prospective study |
| Macias 2015 | Low-Level Laser Therapy at 635 nm for Treatment of Chronic Plantar Fasciitis: A Placebo-Controlled, Randomized Study |

| | |
|---|---|
| Marks 2013 | Low-energy extracorporeal shock-wave therapy in treatment of painful heel: double blind randomized controlled, prospectivetrial with follow-up after 24 months |
| Molund 2018 | Proximal Medial Gastrocnemius Recession and Stretching Versus Stretching as Treatment of Chronic Plantar Heel Pain |
| Monto 2014 | Platelet-Rich Plasma Efficacy Versus Corticosteroid Injection Treatment for Chronic Severe Plantar Fasciitis |
| Niewald 2012 | Randomized, Multicenter Trial on the Effect of Radiation Therapy on Plantar Fasciitis (Painful Heel Spur) Comparing a Standard Dose With a Very Low Dose: Mature Results After 12 Months' Follow-Up |
| Niewald 2015 | Randomized multicenter follow-up trial on the effect of radiotherapy on painful heel spur (plantar fasciitis) comparing two fractionation schedules with uniform total dose: first results after three months' follow-up |
| Ogden 2004 | Electrohydraulic high-energy shock-wave treatment for chronic plantar fasciitis |
| Oliveira 2015 | Effectiveness of total contact insoles in patients with plantar fasciitis |
| Ordahan 2018 | The effect of high-intensity versus low-level laser therapy in the management of plantar fasciitis: a randomized clinical trial |
| Othman 2015 | Endoscopic plantar fasciotomy versus injection of platelet-rich plasma for resistant plantar fasciopathy |
| Ott 2013 | Radiotherapy for calcaneodynia Results of a single center prospective randomized dose optimization trial |
| Peterlein 2012 | Is Botulinum Toxin A Effective for the Treatment of Plantar Fasciitis? |
| Porter 2005 | Intralesional corticosteroid injection versus extracorporeal shock wave therapy for plantar fasciopathy |
| Powell 1998 | Effective treatment of chronic plantar fasciitis with dorsiflexion night splints: A crossover prospective randomized outcome study |
| Radford 2006 | Effectiveness of low-Dye taping for the short-term treatment of plantar heel pain: a randomised trial |
| Radwan 2012 | Resistant plantar fasciopathy: shock wave versus endoscopic plantar fascial release |
| Rahbar 2018 | A Comparison of the Efficacy of Dry-Needling and Extracorporeal Shockwave Therapy for Plantar Fasciitis: A Randomized Clinical Trial |
| Ratna 2015 | Effect of Kinesio Taping in Adjunct to Conventional Therapy in Reducing Pain and Improving Functional Ability in Individuals with Plantar Fasciitis - a Randomized Controlled Trial |
| RenanOrdine 2010 | The effects of calf muscle trigger-points disactivation in plantar fasciitis |
| Riel 2018 | The effect of isometric exercise on pain in individuals with plantar fasciopathy: a randomised crossover trial [with consumer summary] |
| Roca 2016 | Comparison of extracorporeal shock wave therapy with botulinum toxin type A in the treatment of plantar fasciitis |
| Rome 2004 | Evaluating the clinical effectiveness and cost-effectiveness of foot orthoses in the treatment of plantar heel pain - A feasibility study |
| Rompe 2002 | Evaluation of low-energy extracorporeal shock-wave application for treatment of chronic plantar fasciitis |
| Ryan 2014 | Comparison of a Physiotherapy Program Versus Dexamethasone Injections for Plantar Fasciopathy in Prolonged Standing Workers: A Randomized Clinical Trial |
| Saba 2016 | Ultrasound-guided versus palpation-guided local corticosteroid injection therapy for treatment of plantar fasciitis |
| Safeer 2017 | Comparison Study on treatment of Plantar Fasciitis of Foot with Local Steroids Injections and Syptomatic Treatment with Analgesics |
| Shah 2014 | A Study on effect of Myofascial Release in Plantar Fascitis |
| Shah 2018 | Effect of Myofascial Release as an Adjunct Treatment to Conventional Physiotherapy in Plantar Fasciitis |
| Shaheen 2010 | Comparison of three different treatment protocols of low-energy radial extracorporeal shock wave therapy for management of chronic plantar fasciitis |
| Sharifian 2018 | Comparison of the Effect of Prefabricated Foot Orthoses on Pain and Quality of Life in Women With Plantar Fasciitis |

| | |
|---|---|
| Shashua 2015 | The Effect of Additional Ankle and Midfoot Mobilizations on Plantar Fasciitis: A Randomized Controlled Trial |
| Shenoy 2016 | Comparison of Effectiveness of Myofascial Release Technique and Cyriax Technique on Pain Response and Flexibility in Patients with Chronic Plantar Fascitis |
| Srivastava 2017 | Comparison between Effectiveness of Iontophoresis and Conventional Therapy in the Management of Plantar Fasciitis |
| Takla 2018 | Clinical effectiveness of multi-wavelength photobiomodulation therapy as an adjunct to extracorporeal shock wave therapy in the management of plantar fasciitis: a randomized controlled trial |
| Tiwari 2013 | Platelet rich plasma therapy: A comparative effective therapy with promising results in plantar fasciitis |
| Tornese 2008 | Comparison of two extracorporeal shock wave therapy techniques for the treatment of painful subcalcaneal spur. A randomized controlled study |
| Tsai 2006 | Plantar fasciitis treated with local steroid injection: comparison between sonographic and palpation guidance |
| Uğurlar 2018 | Effectiveness of Four Different Treatment Modalities in the Treatment of Chronic Plantar Fasciitis During a 36-Month Follow-Up Period: a Randomized Controlled Trial |
| Vahdatpour 2016 | Autologous platelet-rich plasma compared with whole blood for the treatment of chronic plantar fasciitis; a comparative clinical trial |
| Vahdatpour 2018 | Enhancement of the Effectiveness of Extracorporeal Shock Wave Therapy with Topical Corticosteroid in Treatment of Chronic Plantar Fasciitis: A Randomized Control Clinical Trial |
| Vahdatpour 2012 | Extracorporeal shock wave therapy in patients with plantar fasciitis. A randomized, placebo-controlled trial with ultrasonographic and subjective outcome assessments |
| Vicenzino 2015 | Orthosis-Shaped Sandals Are as Efficacious as In-Shoe Orthoses and Better than Flat Sandals for Plantar Heel Pain: A Randomized Control Trial |
| Winemiller 2003 | Effect of magnetic vs sham-magnetic insoles on plantar heel pain: a randomized controlled trial |
| Yan 2014 | [Therapeutic effect of extracorporeal shock wave combined with orthopaedic insole on plantar fasciitis] |
| Ye 2015 | A comparative efficacy evaluation of ultrasound-guided pulsed radiofrequency treatment in the gastrocnemius in managing plantar heel pain: a randomized and controlled trial |
| Yucel 2010 | Comparison of high-dose extracorporeal shockwave therapy and intralesional corticosteroid injection in the treatment of plantar fasciitis |
| Yüzer 2006 | Comparison of the effectiveness of laser therapy and steroid injection in epin calcanei |
| Zamani 2014 | Comparing the effect of low-power laser therapy with methylprednisolone injection in unilateral plantar fasciitis |
| Zelen 2013 | Prospective, Randomized, Blinded, Comparative Study of Injectable Micronized Dehydrated Amniotic/Chorionic Membrane Allograft for Plantar Fasciitis-A Feasibility Study |
| Zhang 2009 | Acupuncture treatment for plantar fasciitis: a randomized controlled trial with six months follow-up |
| Kamonseki 2016 | Effect of stretching with and without muscle strengthening exercisesfor the foot and hip in patients with plantar fasciitis: A randomizedcontrolled single-blind clinical trial |
| √ñzdemir 2002 | The results of non-invasive conservative treatment of heel pain and the role of subcalcaneal spur in heel pain |
| AguiarGonçalves 2017 | Static, dynamic balance and functional performance in subjects with and without plantar fasciitis |
| Aloulou 2013 | What are the different medical treatment methods for plantar fasciitis? |
| Alvarez 2002 | Preliminary results on the safety and efficacy of the OssaTron (R) for treatment of plantar fasciitis |
| Aranda 2014 | Plantar Fasciitis and Its Relationship with Hallux Limitus |
| Arist√≠nGonz√°lez 2005 | Utility of the radial shock waves in tendon pathology |
| Arslan 2016 | Treatment of Chronic Plantar Heel Pain With Radiofrequency Neural Ablation of the First Branch of the Lateral Plantar Nerve and Medial Calcaneal Nerve Branches |
| Badakhshi 2014 | Low dose radiotherapy for plantar fasciitis. Treatment outcome of 171 patients |

| | |
|---|---|
| Barnes 2017 | Clinical and Functional Characteristics of People With Chronic and Recent-Onset Plantar Heel Pain |
| Berlet 2002 | A prospective trial of night splinting in the treatment of recalcitrant plantar fasciitis: the Ankle Dorsiflexion Dynasplint |
| Beyzadeoglu 2007 | [The effectiveness of dorsiflexion night splint added to conservative treatment for plantar fasciitis] |
| Blanco 2001 | Endoscopic treatment of calcaneal spur syndrome: A comprehensive technique |
| Braun 2018 | Should I Stay or Should I Go? A Prospective, Blinded Study Comparing the Diagnostic Capability of Dynamic and Stationary Pedobarography in Plantar Fasciitis |
| Chang 2007 | Medial calcaneal neuropathy is associated with plantar fasciitis |
| Chang 2012 | Use of MRI for volume estimation of tibialis posterior and plantar intrinsic foot muscles in healthy and chronic plantar fasciitis limbs |
| Chen 2012 | Correlation between computerised findings and Newman's scaling on vascularity using power Doppler ultrasonography imaging and its predictive value in patients with plantar fasciitis |
| Chen 2014 | The "Bodily Pain" Scale of the Short Form-36 Questionnaire is a Predictor of Outcome in Patients who Receive Ultrasound-Guided Corticosteroid Injection for Plantar Fasciitis-A Preliminary Study |
| Chia 2009 | Comparative Trial of the Foot Pressure Patterns between Corrective Orthotics, Formthotics, Bone Spur Pads and Flat Insoles in Patients with Chronic Plantar Fasciitis |
| Concerto 2016 | Anodal transcranial direct current stimulation for chronic pain in the elderly: a pilot study |
| Cotchett 2016 | Depression, Anxiety, and Stress in People With and Without Plantar Heel Pain |
| Cotchett 2017 | The association between pain catastrophising and kinesiophobia with pain and function in people with plantar heel pain |
| Doshi 2016 | Short-term Effects of Kinesiotaping on Pain and Function in Patients with Plantar Fasciitis |
| ElShazly 2010 | Endoscopic plantar fascia release by hooked soft-tissue electrode after failed shock wave therapy |
| Erken 2014 | Prospective Study of Percutaneous Radiofrequency Nerve Ablation for Chronic Plantar Fasciitis |
| Fabrikant 2011 | Plantar fasciitis (fasciosis) treatment outcome study: plantar fascia thickness measured by ultrasound and correlated with patient self-reported improvement |
| Filippou 2004 | Sport related plantar fasciitis. Current diagnostic and therapeutic advances |
| Fleischer 2015 | Prognostic Value of Diagnostic Sonography in Patients With Plantar Fasciitis |
| Gamba 2018 | Relationship of Plantar Fascia Thickness and Preoperative Pain, Function, and Quality of Life in Recalcitrant Plantar Fasciitis |
| Ghafoor 2016 | Effectiveness of Manual Physical Therapy in Treatment of Plantar Fasciopathy |
| Ghandour 2015 | Evaluation and results of modified deep fascial endoscopic plantar fasciotomy |
| Granado 2018 | Metatarsophalangeal joint extension changes ultrasound measurements for plantar fascia thickness |
| Gulick 2000 | Effects of acetic acid iontophoresis on heel spur reabsorption |
| Gupta 2016 | Autologous platelet-rich plasma injection in tennis elbow and plantar fasciitis |
| Harty 2005 | The role of hamstring tightness in plantar fasciitis |
| Hassan 2009 | Percutaneous fenestration of the anteromedial aspect of the calcaneus for resistant heel pain syndrome |
| Hsu 2013 | Effect of shockwave therapy on plantar fasciopathy. A biomechanical prospective |
| Hsu 2018 | Effect of Extracorporeal Shockwave Therapy on Passive Ankle Stiffness in Patients With Plantar Fasciopathy |

| | |
|---|---|
| Ieong 2013 | Ultrasound scanning for recalcitrant plantar fasciopathy. Basis of a new classification |
| Jakobeit 2002 | Ultrasound guided extracorporeal shock wave therapy of symptomatic plantar calcaneal spur |
| Jamali 2004 | Windlass taping technique for symptomatic relief of plantar fasciitis |
| Kapoor 2010 | Realtime elastography in plantar fasciitis: Comparison with ultrasonography and MRI |
| Karabay 2007 | Ultrasonographic evaluation in plantar fasciitis |
| Kayhan 2011 | Sonographically Guided Corticosteroid Injection for Treatment of Plantar Fasciosis |
| Krishnan 2012 | Evaluation of therapeutic effects of extracorporeal shock wave therapy in resistant plantar fasciitis patients in a tertiary care setting |
| Kumar 2013 | The treatment of intractable plantar fasciitis with platelet-rich plasma injection |
| Kuwada 2011 | A prospective randomized trial using four treatment modalities for the treatment of plantar fasciitis |
| Kuyucu 2015 | The association of calcaneal spur length and clinical and functional parameters in plantar fasciitis |
| Li 2013 | [Arthroscopic treatment of painful heel syndrome with radio-frequency] |
| Li 2018 | 3D printing individualized heel cup for improving the self-reported pain of plantar fasciitis |
| Looney 2011 | Graston Instrument Soft Tissue Mobilization and Home Stretching for the Management of Plantar Heel Pain: A Case Series |
| Lorkowski 2009 | [Underfoot pressure distribution of female patients with obesity and plantar fasciitis] |
| Maffulli 2018 | Extracorporeal shock wave therapy in the management of insertional plantar fasciitis: the ASSERT database |
| Mahowald 2011 | The Correlation Between Plantar Fascia Thickness and Symptoms of Plantar Fasciitis |
| Maier 1999 | Castor oil decreases pain during extracorporeal shock wave application |
| Maier 2000 | Extracorporeal shock wave application for chronic plantar fasciitis associated with heel spurs: Prediction of outcome by magnetic resonance imaging |
| Maki 2015 | Correlation between the outcome of extracorporeal shockwave therapy and pretreatment MRI findings for chronic plantar fasciitis |
| McClinton 2015 | Predictors of Response to Physical Therapy Intervention for Plantar Heel Pain |
| McClinton 2016 | Impaired Foot Plantar Flexor Muscle Performance in Individuals With Plantar Heel Pain and Association With Foot Orthosis Use |
| Mehlhorn 2014 | A dorsal night splint with continuous extension of the big toe for treatment of plantar heel pain |
| Meier 2000 | Analgetic effect of extracorporeal shockwaves used for tendinosis calcarea, epicondylitis humeri radialis and plantar fasciitis |
| Melegati 2002 | The influence of local steroid injections, bodyweight and the length of symptoms in the treatment of painful subcalcaneal spurs withextracorporeal shock wave therapy |
| Menz 2019 | Coexistence of plantar calcaneal spurs and plantar fascial thickening in individuals with plantar heel pain |
| Metzner 2010 | High-Energy Extracorporeal Shock-Wave Therapy (ESWT) for the Treatment of Chronic Plantar Fasciitis |
| Miszczyk 2003 | [Evaluation of the effectiveness of the calcaneal spurs radiotherapy] |
| Moyne-Bressand 2018 | Effectiveness of Foot Biomechanical Orthoses to Relieve Patients Suffering from Plantar Fasciitis: Is the Reduction of Pain Related to Change in Neural Strategy? |
| Nakale 2018 | Association Between Plantar Fasciitis and Isolated Gastrocnemius Tightness |

| | |
|---|---|
| Onwuanyi 2000 | Calcaneal spurs and plantar heel pad pain |
| Osborne 2006 | Critical differences: in lateral X-rays with and without a diagnosis of plantar fasciitis |
| Park 2014 | Long-term outcome of low-energy extracorporeal shock wave therapy for plantar fasciitis: comparative analysis according to ultrasonographic findings |
| Perlick 1998 | High-energy extracorporal shock wave therapy in plantar calcaneal spur |
| Placzek 2005 | [Botulinum toxin A--therapy option in cases of chronic plantar fasciitis?--an open treatment attempt with 9 patients and a one year observation period] |
| Placzek 2006 | Treatment of chronic plantar fasciitis with Botulinum toxin A - An open pilot study on 25 patients with a 14-week-follow-up |
| Prasetyo 2017 | Additional diagnostic value of digital radiology in plantar fasciitis diagnosis |
| Putz 2017 | Investigation of the acute plantar fasciitis with contrast-enhanced ultrasound and shear wave elastography - first results |
| Rano 2001 | Correlation of heel pain with body mass index and other characteristics of heel pain |
| Reeboonlap 2012 | Outcome of plantar fasciitis treatment using monochrome infrared irradiation |
| Riddle 2003 | Risk factors for plantar fasciitis: A matched case-control study |
| Riel 2019 | Translation and cultural adaptation of a Danish version of the Foot Health Status Questionnaire for individuals with plantar heel pain |
| Ring 2014 | Clinical efficacy and cost-effectiveness of bespoke and prefabricated foot orthoses for plantar heel pain: a prospective cohort study |
| Rome 2001 | Generalised joint hypermobility: a poor predictor of plantar fasciitis in athletes |
| Rose 2003 | Neurosensory testing of the medial calcaneal and medial plantar nerves in patients with plantar heel pain |
| Sabir 2005 | Clinical utility of sonography in diagnosing plantar fasciitis |
| Scheuer 2016 | Approaches to optimize focused extracorporeal shockwave therapy (ESWT) based on an observational study of 363 feet with recalcitrant plantar fasciitis |
| Sean 2010 | Radiofrequency microtenotomy for the treatment of plantar fasciitis shows good early results |
| Serviat-Hung 2015 | [Extracorporeal shockwave terapy in sports and non-sports population. Preliminary results] |
| Sistermann 1998 | Complications, side effects and contraindications using middle and high energetic extracorporeal shock waves in orthopaedics |
| Sorensen 2011 | Percutaneous Bipolar Radiofrequency Microdebridement for Recalcitrant Proximal Plantar Fasciosis |
| Stropek 2008 | Arthroscopic Treatment for Calcaneal Spur Syndrome |
| Sutera 2010 | Plantar fascia evaluation with a dedicated magnetic resonance scanner in weight-bearing position: our experience in patients with plantar fasciitis and in healthy volunteers |
| Tay 2012 | Open technique is more effective than percutaneous technique for TOPAZ radiofrequency coblation for plantar fasciitis |
| Turgut 1999 | The relationship of heel pad elasticity and plantar heel pain |
| Uzel 2006 | Comparison of ultrasonography and radiography in assessment of the heel pad compressibility index of patients with plantar heel pain syndrome. Measurement of the fat pad in plantar heel pain syndrome |
| Valizadeh 2018 | Relationship Between Anthropometric Findings and Results of Corticosteroid Injections Treatment in Chronic Plantar Heel Pain |
| VanTonder 2018 | Multidimensional impact of low-Dye taping on low-load hopping in individuals with and without plantar fasciitis |
| Vetrano 2014 | Cross-cultural adaptation and reliability of the Italian version of the Foot Function Index (FFI-I) for patients with plantar fasciitis |
| Walther 2004 | Power doppler findings in plantar fasciitis |

| | |
|---|---|
| Wearing 2004 | Sagittal movement of the medial longitudinal arch is unchanged in plantar fasciitis |
| WeilJr 2008 | A new minimally invasive technique for treating plantar fasciosis using bipolar radiofrequency: a prospective analysis |
| Wheeler 2013 | Autologous blood injections for chronic plantar fasciitis - a pilot case-series study shows promising results |
| Wheeler 2018 | Extracorporeal Shockwave Therapy Plus Rehabilitation for Patients With Chronic Plantar Fasciitis Might Reduce Pain and Improve Function but Still Not Lead to Increased Activity: A Case-Series Study With Multiple Outcome Measures |
| Wu 2008 | Reliability and validity of the Taiwan Chinese version of the foot function index |
| Wu 2011 | Sonoelastography of the Plantar Fascia |
| Wu 2013 | [Case-control study of stretching exercise on treatment of plantar fasciitis] |
| Wu 2015 | Plantar fascia softening in plantar fasciitis with normal B-mode sonography |
| Yadav 2012 | Comparison of the effects of therapeutic ultrasound v/s myofascial release technique in treatment of plantar fasciitis |
| Yildiz 2018 | Changes in Rearfoot Alignment in Chronic Plantar Heel Pain |
| Yoosefinejad 2015 | Effects of Extracorporeal Shock Wave Therapy on Numerical Rating Scale of Pain in Patients with Chronic Plantar Fasciitis |
