# Supplementary material for: Large variation in participant eligibility criteria used in plantar heel pain research studies - a systematic review
Source: J Foot Ankle Res. 2022 Sep 9;15:69. doi: 10.1186/s13047-022-00573-0 (PMC9461187; doi:10.1186/s13047-022-00573-0)
Supplement: Supplementary file 2 — Additional file 2. [file 13047_2022_573_MOESM2_ESM.pdf]

BMI			
**Criterion**	**Weighted mean (kg/m²)**	**Number of studies reporting characteristic**	**Number of participants**
No	29.2	103	6119
Between 18 and 30	24.4	1	30
Between 18 and 32	29.1	1	40
Between 20 and 30	29.5	2	174
Less than 29.2	26.2	1	70
Less than 30	26.9	2	168
Less than 35	28.9	2	91
Less than 40	30	1	10

Pain			
**Criterion**	**Weighted mean (0 to 10 VAS)**	**Number of studies reporting characteristic**	**Number of participants**
No	6.9	94	5144
≥2	6.0	2	50
≥3	4.1	3	237
Between 3 and 8	6.3	1	30
≥4	6.0	2	60
≥4.9	7.8	1	36
≥5	6.1	3	114
≥6	8.9	3	282

Symptom duration			
**Criterion**	**Weighted mean (months)**	**Number of studies reporting characteristic**	**Number of participants**
No	16.2	53	3766
≥1 week	17.9	1	142
≥1 month	40.3	12	914
≥6 weeks	4.1	4	266
Between 2 and 12 months	4.8	1	45
≥2 months	5.2	4	211
≥3 months	16	12	452
≥4 months	8.8	2	65
≥6 months	16.2	20	1443
<12 months	5.5	1	63
≥12 months	39.4	3	179

Age			
**Criterion**	**Weighted mean (years)**	**Number of studies reporting characteristic**	**Number of participants**
No	49.2	100	5803
Between 15 and 60	39.7	1	29
Between 18 and 60	45.7	4	251
Between 18 and 65	46.6	4	201
Between 18 and 70	45	2	72
Between 18 and 75	51.5	2	74
Between 18 and 80	58	1	10
Between 20 and 50	42.1	2	95
Between 20 and 68	49.6	1	40
Between 20 and 70	36.3	2	82
Between 30 and 60	44.9	1	8
Between 30 and 62	54.5	1	30
Between 38 and 75	54.5	1	84
Between 40 and 60	48.9	3	90

≥15	52.4	1	72
≥18	49	55	3764
≥20	54.1	2	56
≥21	49.8	1	20
≥40	56.4	3	204
≥50	64.3	1	117
≥65	70.1	3	246
Skeletally mature	46.7	2	52
